# A Review on Root Anatomy and Canal Configuration of the Maxillary Second Molars

**DOI:** 10.22037/iej.2017.01

**Published:** 2017

**Authors:** Negin Ghasemi, Saeed Rahimi, Shahriar Shahi, Mohammad Samiei, Mohammad Frough Reyhani, Bahram Ranjkesh

**Affiliations:** a *Dental and Periodontal Research Center, Department of Endodontics, Dental School, Tabriz University of Medical Sciences, Tabriz, Iran; *; b *Department of Endodontics, Dental School, Tabriz University of Medical Sciences, Tabriz, Iran; *; c * Department of Dentistry and Oral Health, Aarhus University, Health, Denmark Section for Dental Pathology, Operative Dentistry and Endodontics, Denmark*

**Keywords:** Maxillary Second Molar, Root Canal Anatomy, Root Morphology, Systematic Review

## Abstract

**Introduction:**

The complexity of the root canal system presents a challenge for the practitioner. This systematic review evaluated the papers published in the field of root canal anatomy and configuration of the root canal system in permanent maxillary second molars.

**Methods and Materials:**

All articles related to the root morphology and root canal anatomy of the permanent maxillary second molars were collected by suitable keywords from PubMed database. The exhaustive search included all publications from 1981 to December 2015. The articles relevant to the study were evaluated and data was extracted. The author/year of publication, country, number of the evaluated teeth, type of study (method of the evaluation), number of roots and the canals, type of canals and the morphology of the apical foramen was noted.

**Results:**

The highest studied populations were in Brazil and United States. A total of 116 related papers were found, which had investigated 11945 teeth in total. Across all the studied populations, the three-rooted anatomy was most common, while the four-rooted anatomy had the lowest prevalence. The presence of the second mesiobuccal canal ranged from 11.53 % to 93.7%, where type II (2-1) configuration was the predominant type in Brazil and USA and types II and III (1-2-1) in Chinese populations. In 8.8-44% of cases, fusion was observed. The main reported cases were related to palatal root. The major method of anatomical investigation in case reports was periapical radiography, and the chief method in morphological studies was CBCT.

**Conclusion:**

The clinicians should be aware of normal morphology and anatomic variations to reduce the treatment failure.

## Introduction

Cleaning, shaping and three-dimensional obturation of the root canal system are the keys to successful endodontic treatment, that requires knowledge of the anatomy of the root canal system [1-3]. However, an important challenge is the complexity of the root canal system and anatomical variations [3]. Therefore, the clinician should be aware of typical configuration and potential anatomical variations. In this case, the possibility of treatment failure due to untreated canals decreases [4]. There are various ways for evaluating the anatomy of the root canal system including preparation of access cavity and radiography while the file is in the root canal. Other methods include canal staining and tooth clearing, conventional and digital radiography, computed tomography (CT), cone-beam computed tomography (CBCT), serial sectioning and microscopic evaluation [5-9]. 

Anatomical variations are possible in every tooth, and the second maxillary molar is no exception [10, 11]. Typically, this tooth has three roots [12]. The mesiobuccal (MB) root of maxillary molars has always been a challenge, holding also true for the second molar [13]. A significant number of studies in many countries have dealt with the anatomical and morphological investigation of the root canal system of this tooth [14-18]. Various case studies have also been published in this regard [2, 19-21]. 

The copious number of articles was published regarding the root canal anatomy of the second maxillary molar most of which, studied populations and the number of examined teeth make the result interpretation difficult and time-consuming. In such cases, review papers can provide valuable information about the normal morphology and different variations present in the root canal system to readers. Since there was not any published review article regarding the root anatomy and canal configuration of the second maxillary molar, this systematic review was conducted on investigations and case studies published regarding the anatomy and morphology of the root canal system of the maxillary second molar.

## Materials and Methods

An exhaustive search was undertaken to identify published literature related to the root anatomy and root canal morphology of the permanent maxillary second molar *via* PubMed database. English papers which evaluated one aspect of root morphology and root canal anatomy of the second maxillary molars were included in this review.

The searched keywords were Maxillary Second Molar, Root Morphology and Root Canal Anatomy. The search included all publications from 1981 to December 2015. Titles and abstracts were evaluated. The articles relevant to the study were evaluated regarding the following data: The author/year of publication, country, number of the evaluated teeth, type of study, number of roots and the canals, type of canals according to Vertucci’s classification and the morphology of the apical foramen.

## Results

In total, 116 papers were found according to the mentioned entry criteria, which had assessed a total number of 11945 teeth. Among these 56 papers were case reports, presented in Table 1 [2, 11, 12, 19-71]. In the majority of earlier studies, the applied method was radiography, whereas in more recent studies, the tendency has been towards CBCT. Twenty tree papers were related to palatal root, most of which involved reporting the presence of two separate palatal roots. 

Among the examined studies, the number of roots of the second maxillary molar was investigated in 10 studies [6, 8, 14, 21, 72-77] (Table 2). In these investigations, three-rooted anatomy claimed the highest percentage, while the four-rooted morphology had the lowest percentage reported among all of the examined teeth. Moreover, CBCT technique was the most utilized method in these studies. As few as 6 studies dealt with root fusion in this tooth [6, 8, 73, 74, 78, 79] (Table 3), with the Brazilian and Iranian populations take the highest and lowest prevalence, respectively. Among the roots of the second maxillary molar, the mesiobuccal root appropriated the largest number of studies, with 33 papers being found in this regard [6, 8, 13, 16, 17, 72, 75, 78-104] (Table 4). 

The presence of the second mesiobuccal canal ranged from 11.53% [105] to 93.7% [99]. The predominant reported canal type was related to the studied populations; where type II (2-1) was the predominant type in Brazil and USA and types II, III (1-2-1) were more prevalent in Chinese populations. The largest number of studies in this regard was conducted in Brazil and USA, where again CBCT method was found in many of the more recent studies. 

Some investigations have evaluated the distance between the orifice of second mesiobuccal and first mesiobuccal canals. In one study, second mesiobuccal canal was located 2.2±0.54 mm palatally and 0.98±0.35 mm mesially, in relation with main mesiobuccal canal [13]. In another study, it was reported to be located 2 mm palatally and 1 mm mesially [8]. 

The two morphological studies on various dimensions of the mesiobuccal root, it was found that there was no difference between the diameter of the wall of the mesial and distal root in the apical and medial one third. However, in the coronal 1/3, the thickness of the distal wall of the root was 33% lower [89, 91]. In the second molar, unlike the first molar, the thickness of the distal wall of mesiobuccal canals in CEJ level and 2 and 4 mm apically than CEJ, was not different [91]. The shape of the pulp chamber floor in one study was rhomboid [106] but in another evaluation was quadrilateral [78]. 

A number of studies also explored the anatomy of distobuccal root. One study, using radiography and decalcification, indicated two canals in the distobuccal root by 4% in the mesiodistal dimension and 6% in the buccolingual dimension [81]. In the morphological study, it was reported that the prevalence of extra canal present in the distobuccal root as 0.3% [6]. In three studies, the presence of one canal in the distobuccal root was 96, 92 and 84.9%, respectively [16, 72, 105]. In one survey, in Chinese population using CBCT, the mean distance between the orifice of mesiobuccal and distobuccal canals was 0.7-4.8 mm, and between palatal and distobuccal was 0.8-6.7 mm [15]. 

Regarding the anatomy of the palatal root, one investigation evaluated 25 teeth by micro-CT method whereby 16 teeth were type I (two palatal roots are very divergent and often long and tortuous, which can be observed radiographically), 7 were type II (the palatal roots are shorter and parallel and root apices are blunt, with mesial and distal divergence on the buccolingual radiographic view) and 2 were type III (the roots have a constricted morphology with mesiobuccal, mesiopalatal and distopalatal roots engaged in a web-like radiographic view similar to type II) were reported [78]. In one research, the prevalence of two canals in the palatal root was reported to be 1.82% [6]. In one anatomic investigation using CBCT, it was stated that 11 out of 979 teeth (1.12%) had two palatal roots, in which gender and the jaw side were not influential [107]. The mean distance between the orifice of the mesiopalatal and distopalatal canals was 2.84±0.5 mm. The angle between two palatal roots was reported to be 34.6±16.1 mm [107]. In an *in vivo* study using CBCT in Chinese population, which investigated 1226 teeth [21]; they found that 12 cases had two palatal roots and the section of the distopalatal canal was larger. The presence of two canals in the palatal root was reported 6% [105] and 12.2% [16].

The symmetry of the second molar has been investigated in two studies [6, 108]. They reported that in 79.6% and 82.7% [109] of studied cases both the right and left molars were symmetric and had three root canals.

In one investigation, the degree of presence of two physiological foramen in the mesiobuccal root was 71.15%. Accessory foramens existed in 33% of cases and in 70% of cases, the foramen was oval shaped. The size of the foramen in the buccal canals ranged from 0.18-0.25%, which was 0.22-0.29 mm in the palatal canal [110]. In another anatomical study regarding the apical foramen, the predominant morphology of foramen and apex has been reported to be round shaped, where in 39.7% and 58.4% of cases, apex and foramen were in the center of the root, respectively [5]. 

In the some morphological studies [72, 73, 78, 111] prevalence of isthmus, apical delta and lateral canal in the mesiobuccal root was greater than in other roots. These cases were more present in apical 1/3. In one anatomical study, the isthmus tissue and 80% of accessory canals were positioned whiten 3.6 mm coronally from the apex [111]. 

The distance between the pulp floor and furcation was evaluated in two articles [78, 112] and was reported to be 3.05±0.9 and 0.57±2.15 mm, respectively. The distance between the buccal cusp and furcation and pulp floor was 11.15±1.21, 0.88±8.08%, respectively. Moreover, the height of the pulp chamber was stated to be 1.8±0.68 mm [112]. 

Presence of C-shaped canal in the second maxillary molar was investigated in some studies and reported about 4.9% for this anatomic variation [18, 113, 114]. Rare anatomical findings were observed in some morphological studies. Prevalence of enamel pearl in one study was 8% [78]. In one investigation in German population, the prevalence of taurodontism and pyramid-shaped molar was reported to be 18/800 and 15/800, respectively [115].

## Discussion

The second maxillary molar has a complex root canal system and one of the reasons of failure in endodontic treatment is lack of locating and cleaning of the entire root canal system [2]. The complexity of the root canal system of the second maxillary molar is largely related to presence of the second mesiobuccal canal [102, 103, 134]. The first report published on the existence of excess canal in the mesiobuccal root of the second maxillary molar is related to the study by Hess and Zurcher in 1925 [135]. In this review study, a considerable number of case and morphological studies have dealt with reporting two canals in the mesiobuccal root. 

Our investigation indicates a difference between the prevalence of the second mesiobuccal canal across several studies, possibly due to the evaluation techniques employed as well as the racial diversities. On the other hand, definition of the second mesiobuccal canal across studies is different. Some researchers have sufficed to stating presence of two individual orifices onto the pulp floor and primary localization [136]. According to Stropko [101], the second canal can be considered as the second mesiobuccal canal if the file can be inserted in the canal by 3-4 mm. More recent studies have considered a more accurate criterion, in which the second mesiobuccal canal is absolutely separate from the first mesiobuccal canal; and before reaching to each other in the apex, they are 5 mm away off each other; also, they should also remain separate from each other following instrumentation [95]. Various factors can affect the finding of an excessive canal like the second mesiobuccal canal. One of these factors is the practitioner's experience; it has been found that great experience of the practitioner helps in locating of the extra canals like MB2 [137].

In this review study, having investigated the papers related to the second mesiobuccal canal, it can be concluded that age is an important factor and has a significant effect on the number of found canals [79, 84, 88]. As the age increases by one, the chance of finding canals drops dramatically 0.98 times, related to calcification and morphological changes occurring by ageing. Further, in a decayed tooth, the possibility of finding an extra canals is 1.4 times greater than in non-decayed teeth [7, 84]. *In vitro* studies, compared to *in vivo* examinations, as well as in retreatment compared with primary treatment, report a higher chance of finding extra canals [95, 96]. Increased chance of finding extra canals with the help of magnification, especially microscope is a common finding across all of the investigated studies [98, 138, 139]. Only Sempira *et al. *[100], have stated that use of microscope is not effective.

The possibility of finding extra canal in the study by Sert and Byirli [140] was related to gender, however in another study, no relationship was found between these two variables [84]. Among the investigated studies, one has stated that there is an inverse relationship between the root zone and finding canal, and as the canal approaches the apical 1/3, the possibility of detection declines [84]. One of the factors highlighting this especially in more recent studies is use of novel imaging techniques such as tomography. Although in the majority of earlier studies, the clearing technique, as the gold standard, has been used. It is an *in vitro* model developed on extracted teeth. The size of samples is limited and lack of possibility of analyzing similar teeth in other quadrants is another flaw of it [141]. It should always be noted that it is still a valuable techniques which is accurate, simple and applicable *in vivo*. In some other studies, typical radiography was used, presenting a two-dimensional image of a three-dimensional object. There is a chance of distortion and superimposition, diminishing the possibility of complex morphological examinations [9]. The CBCT technique, as a variation of computer tomography, provides the possibility of three-dimensional understanding of morphology and high resolution with a low radiographic dose [9, 74, 86].

**Table 1:** Case reports on maxillary second molars

**Authors**	**Type of study**	**Number of teeth**	**Description**
**Beshkenadze and Chipashvili**	*In vivo *(PA radiographs)	2	2 roots, 2 canals, 3 roots, 4 canals
**Chawala ** ***et al.***	*In vivo *(CBCT)	1	6 canals, 2 in M, 2 in D, 2 in P
**Hans ** ***et al.***	*In vivo *(PA radiographs)	2	Microdontia
**Jaikrishan ** ***et al.***	*In vivo *(CBCT)	2	1 root and 1 canal
**Radwan and Kim**	*In vivo* (PA radiographies+CBCT)	2	Hyper taurodontism
**Ahmad and Al-jadda**	*In vivo *(PA radiographs)	2	2 roots, 2 canals, 3 roots,4 canals
**Shah ** ***et al.***	*In vivo *(PA radiographs)	1	2 canals in MB root
**Ashraf ** ***et al.***	*In vivo*(CBCT)	1	2 roots,4 canals, (2 M canals, 2 D canals)
**Fakhari and Shokraneh**	*In vivo *(PA radiographies+flap)	1	2 canals in P
**Paul ** ***et al.***	*In vivo *(PA radiographs)	1	2 independent P roots
**Brito ** ***et al.***	*In vivo *(loup+DOM+CBCT)	1	3 B roots and midbuccal canal
**Simsek ** ***et al.***	*In vivo *(CBCT)	1	2 roots, 4 canals, (2 M canals, 2 D canals)
**Arora ** ***et al.***	*In vivo *(MDCT)	1	3 canals in MB roots
**Eskandarinekhad and Ghasemi**	*In vivo *(PA radiographies+loup)	1	2 roots, 4 canals, (2 in P, 2 in B)
**Shojaeian ** ***et al.***	*In vivo *(PA radiographs)	1	2 P canals, Enamel pearl
**Patel and Patel**	*In vivo *(PA radiographs)	2	2 canals in P
**Ioannidis ** ***et al.***	*In vivo *(CBCT)	2	One root, one canal
**Scarparo ** ***et al.***	*In vivo *(PA radiographs)	5	2 canals in P root
**Zhu and Zhao**	*In vivo* (CT)	1	3 canals in MB root
**Zha ** ***et al.***	*In vivo *(PA radiographs)	1	5 canals, 2 in M, 2 in D, 2 in P
**Wang ** ***et al.***	*In vivo *(CBCT)	1	one root, one canal
**Crincoli ** ***et al.***	*In vivo *(micro radiograph)	1	Dens invagination
**Singla and Aggarwal**	*In vivo* (spiral CT)	1	C-shaped P Canal
**Weinstein ** ***et al.***	*In vivo *(endoscope)	1	Gemination
**Prashanth ** ***et al.***	*In vivo *(PA radiographs)	1	2 palatal canals
**Morinaga ** ***et al.***	*In vivo* (PA radiographs)	1	Dens invagination

**Table 2:** Number of roots in maxillary second molars

**Authors**	**Number of teeth**	**Country**	**Type of the study**	**1 root**	**2 roots**	**3 roots**	**4 roots**
**Zhang ** ***et al.***	210	China	CBCT	10%	8%	81%	
**Rweuyonyi ** ***et al.***	221	Ugandan	clearing			86%	
**Ng ** ***et al.***	77	London	clearing			100%	
**Gu ** ***et al.***	1226	China	CBCT				98%
**Rouhani ** ***et al.***	125	Iran	CBCT				1.6%
**Georgia ** ***et al.***	402	Greek	CBCT	5.4%	8.25%	85.07%	1.2%
**Silva ** ***et al.***	306	Brazil	CBCT			45.09%	
**Libfeld**	1200	Israel	Radiography/RCT	3%, 0.5%	6%, 12%	90.6%, 87%	0.4%
**Kim ** ***et al.***	821	Korea	CBCT	4.63%			
**Peikoff ** ***et al*** **.**	520	Canada	Radiography	3.1%	6.9%	80.5%	1.4%

**Table 3:** Fusion in maxillary second molar

**Authors**	**Number of teeth**	**Country**	**Type of the study**	**Fusion**
**Versiani ** ***et al.***	25	Brazil	RCT	44%
**Kim ** ***et al.***	821	Korea	CBCT	10.71%
**Zhang ** ***et al.***	187	China	RCT	42.25% 22 partial-6 complete merge)
**Rouhani ** ***et al.***	125	Iran	CBCT	8.8%
**Rwenyonyi ** ***et al.***	221	Ugandan	Clearing	13.1% (MB with DB: 6.8% -MB with P: 6.3%)
**Al-shalabi ** ***et al.***	40	Irland	Clearing	43%

**Table 4:** Mesiobuccal root canal system configuration

Author	Number of teeth	Country	Type of study	Prevalence of MB2 canal
Betancourt *et al.*	225	Chile	*In vivo* (CBCT)	48%
Singh *et al.*	100	India	*In vitro* (clearing)	19.4% Type II:15.3% Type IV:2.7% Type V:1.4%
Silva *et al.*	306	Brazil	*In vivo* (CBCT)	34/32%
Li *et al.*	50	China	*In vitro* (CBCT)	41.3%, Type I: 54.4%
Al-Fouzan *et al.*	162	Saudi Arabia	*In vivo* (radiography)	19.7%
Domark *et al.*	14	USA	*In vitro*(CBCT, Digital RG)	57%
Reis *et al.*	185	Brazil	*In vivo* (CBCT)	Right molars 87.5% Left molars 79.3%
Silveria *et al.*	43	Brazil	*In vitro* (CBCT,DOM)	Negotiable 80.2%-81.4%
Vizzotto *et al.*	89	Brazil	*In vitro* (CBCT)	67%
Versiani *et al.*	25	Brazil	*In vitro* (micro CT)	Type I :16 Type 2:7 Type 3: 2
Kim *et al.*	821	Korea	*In vivo* (CBCT)	34/39%
Bauman *et al.*	12	USA	*In vitro* (CBCT)	92%
Zhang *et al.*	210	China	*In vivo* (CBCT)	22% Type II:18% Type IV:58% Type V:10% Type VI:3%
Lee *et al.*	467	Korea	*In vivo* (CBCT)	42.2%, Mainly Wien's type II and III
Neelakatan *et al.*	205	India	*In vitro* (CBCT)	50%
Degerness and Bowles	63	USA	*In vitro*(Serial Section and stereomicroscope)	60.3%
Zhao *et al.*	118	China	*In vitro* (RG)	49.15% Type I:46.30% Type II:12.96% Type III:31.48%
Gao *et al.*	334	China	*In vitro* clearing+spiral CT scanning)	49.70%
Xoshioka *et al.*	208	Korea	*In vivo* DOM and troughing	48%
Walcott *et al.*	2038	USA	*In vivo* (RCT and radiography)	35% Initial treatment 34% Retreatmentn40%
Wang	52	China	*In vivo* (RCT and radiography)	11.53% Negotiable 7.69%
Zhang *et al.*	113	China	*In vitro* (OM)	52.2% Negotiable64.3%
Wolcott *et al.*	680	USA		Initial treatment 35% Retreatment 44%
Buhrley *et al.*	104	USA	*In vivo* (Loup, DOM)	Without magnification 20% Loup40.5% DOM36.1%
Schwarze *et al.*	50	Germany	Loup, DOM,sectioning	24.6% (section) 41.1%(loup) 93.7% (DOM)
Ng *et al.*	77	UK	*In vitro* (clearing)	49% (canal type mainly II and IV)
Sempira and Hartwell	100	USA	*In vivo* (DOM)	Negotiable 24.3%
Al-Shalabi *et al.*	40	Ireland	*In vitro* (clearing)	58% (mainly type IV)
Stropko *et al.*	611	USA	*In vivo *(clinical RCT with DOM)	45.6%
Eskoz and Weine	73	USA	*In vitro *(Radiography)	41.3% Type II 20.9% Type III 16.4% Type IV 3%
Singh *et al.*	50	Punjab	*In vitro*(decalcification)	78% in MD and 20% in BL direction
Pecora *et al.*	200	Brazil	*In vitro* (clearing)	42%
Gilles and Header	37	Columbus	*In vitro* (SEM)	70%

Examination of the papers evaluating various techniques for finding the second mesiobuccal canal indicates that there is not any difference between CT and CBCT, but both methods are better than digital radiography [83]. There is no significant difference between CT and CBCT in comparison with serial sectioning and clearing, either [4, 83, 90]. In another study, the results of CBCT and transparent tooth technique were congruent [80]. In a study regarding voxel size in CBCT, 0.3 mm was stated as suitable for CBCT [86]. In another study with a voxel size of 0.4 mm, the reliability of detection was 60.1% and with a voxel size of 0.125, was reported to be 93.3% [87]. 

In the majority of studies, the significance of utilizing magnification especially microscope has been underscored [85, 98, 99, 103, 139]. However, as found by Sempira and Hartwell [100], there is no difference between the ability of finding the second mesiobuccal canal in those in which access cavity has been modified with no microscope in comparison with presence of microscope. 

In the conducted studies, it has been emphasized that removal of the obturation materials from the canal resulted in better detection of the extra canals and morphological complexities by this method [4, 86, 142]. On the other hand, this method is suitable in detecting the mapping of canals, rather than detecting the negotiability of the canal [85]. CBCT is not usable for a tooth in typical clinical practice.

In a study, it was reported that the CMOS (complementary metal oxide semiconductor) imaging technology enhanced reliability of the second mesiobuccal canal detection and when radiography is of interest, it has an optimal exposure [143]. 

Another point mentioned with regard to the second mesiobuccal canal was the negotiability of the found orifice. A number of studies, in addition to examining the extent of MB2 canal, evaluated its negotiability as well [85, 97, 100]. Aggregation of the dentin debris and other debris produced through pathfinding, presence of anatomical variations, diffused calcification of the pulp and presence of pulp stone are factors influencing the negotiation of the canal [144]. 

To have a successful canal treatment in the second maxillary molar, cleaning should not focus only on the second mesiobuccal canal and mesiobuccal root. Investigation of the studies published on the morphology of this tooth indicates that anatomical variations are also present considerably in palatal root (Table 1), where presence of two canals has been the most reported case. However, the distobuccal canal should not be overlooked.

Anatomical landmarks, the dimensions of the pulp chamber together with the thickness of root walls, presence of isthmii and peripheral canals, as well as the size and position of the apical foramen have also been taken into consideration in a limited number of studies [5, 89, 106, 145]. These studies were valuable because of reducing the probability of perforation and gouging during treatment and enhancing cleansing the entire pulp system.

## Conclusion

The complexity of the canal system is influenced by genetics and this factor should be considered before interpreting and comparing the results of various morphological studies, in addition to factors like age and gender.

## References

[1] Shakouie S, Mokhtari H, Ghasemi N, Gholizadeh S (2013). Two-rooted maxillary first molars with two canals: a case series. Iran Endod J.

[2] Eskandarinezhad M, Ghasemi N (2012). Nonsurgical endodontic retreatment of maxillary second molar with two palatal root canals: a case report. J Dent Res Dent Clin Dent Prospects.

[3] Rahimi S, Ghasemi N (2013). Maxillary first molar with two root canals. Sultan Qaboos Univ Med J.

[4] Blattner TC, George N, Lee CC, Kumar V, Yelton CD (2010). Efficacy of cone-beam computed tomography as a modality to accurately identify the presence of second mesiobuccal canals in maxillary first and second molars: a pilot study. J Endod.

[5] Martos J, Lubian C, Silveira LF, Suita de Castro LA, Ferrer Luque CM (2010). Morphologic analysis of the root apex in human teeth. J Endod.

[6] Kim Y, Lee SJ, Woo J (2012). Morphology of maxillary first and second molars analyzed by cone-beam computed tomography in a korean population: variations in the number of roots and canals and the incidence of fusion. J Endod.

[7] Iqbal M, Fillmore E (2008). Preoperative predictors of number of root canals clinically detected in maxillary molars: a PennEndo Database study. J Endod.

[8] Zhang R, Yang H, Yu X, Wang H, Hu T, Dummer PM (2011). Use of CBCT to identify the morphology of maxillary permanent molar teeth in a Chinese subpopulation. Int Endod J.

[9] Neelakantan P, Subbarao C, Subbarao CV (2010). Comparative evaluation of modified canal staining and clearing technique, cone-beam computed tomography, peripheral quantitative computed tomography, spiral computed tomography, and plain and contrast medium-enhanced digital radiography in studying root canal morphology. J Endod.

[10] Alrahabi M, Sohail Zafar M (2015). Evaluation of root canal morphology of maxillary molars using cone beam computed tomography. Pak J Med Sci.

[11] Benenati FW (1985). Maxillary second molar with two palatal canals and a palatogingival groove. J Endod.

[12] Kottoor J, Hemamalathi S, Sudha R, Velmurugan N (2010). Maxillary second molar with 5 roots and 5 canals evaluated using cone beam computerized tomography: a case report. Oral Surg Oral Med Oral Pathol Oral Radiol Endod.

[13] Betancourt P, Navarro P, Cantin M, Fuentes R (2015). Cone-beam computed tomography study of prevalence and location of MB2 canal in the mesiobuccal root of the maxillary second molar. Int J Clin Exp Med.

[14] Georgia NE, Taxiarchis KG, Nikolaos KP (2015). Evaluation of the Root and Canal Morphology of Maxillary Permanent Molars and the Incidence of the Second Mesiobuccal Root Canal in Greek Population Using Cone-beam Computed Tomography. Open Dent J.

[15] Han X, Yang H, Li G, Yang L, Tian C, Wang Y (2012). A study of the distobuccal root canal orifice of the maxillary second molars in Chinese individuals evaluated by cone-beam computed tomography. J Appl Oral Sci.

[16] Neelakantan P, Subbarao C, Ahuja R, Subbarao CV, Gutmann JL (2010). Cone-beam computed tomography study of root and canal morphology of maxillary first and second molars in an Indian population. J Endod.

[17] Pecora JD, Woelfel JB, Sousa Neto MD, Issa EP (1992). Morphologic study of the maxillary molars Part II: Internal anatomy. Braz Dent J.

[18] Fernandes M, de Ataide I, Wagle R (2014). C-shaped root canal configuration: A review of literature. J Conserv Dent.

[19] Fakhari E, Shokraneh A (2013). A maxillary second molar with two separate palatal roots: a case report. J Dent (Shiraz).

[20] Fava LR, Weinfeld I, Fabri FP, Pais CR (2000). Four second molars with single roots and single canals in the same patient. Int Endod J.

[21] Gu Y, Wang W, Ni L (2015). Four-rooted permanent maxillary first and second molars in a northwestern Chinese population. Arch Oral Biol.

[22] Alani AH (2003). Endodontic treatment of bilaterally occurring 4-rooted maxillary second molars: case report. J Can Dent Assoc.

[23] Arora A, Acharya SR, Saraswathi MV, Sharma P, Ather A (2013). Dilemmas pertaining to three canals in the mesiobuccal root of a maxillary second molar: a case report. Restor Dent Endod.

[24] Asgary S (2007). Endodontic treatment of a maxillary second molar with developmental anomaly: a case report. Iran Endod J.

[25] Asghari V, Rahimi S, Ghasemi N, Talebzadeh B, Norlouoni A (2015). Treatment of a Maxillary First Molar with Two Palatal Roots. Iran Endod J.

[26] Ashraf H, Dianat O, Hajrezai R, Paymanpour P, Azadnia S (2014). Endodontic treatment of a double-rooted maxillary second molar with four canals: a case report. Iran Endod J.

[27] Barbizam JV, Ribeiro RG, Tanomaru Filho M (2004). Unusual anatomy of permanent maxillary molars. J Endod.

[28] Beshkenadze E, Chipashvili N The maxillary second molar - anatomical variations (case report). Georgian Med News.

[29] Brenna F, Benanini M, Vescovi P, Frigeri S (1990). [Therapy of a maxillary second molar with 5 root canals]. Attual Dent.

[30] Chawla A, Sujlana A, Dixit A (2015). Re-treating a maxillary second molar with 6 root canals assisted by cone beam computed tomography. Gen Dent.

[31] Christie WH, Peikoff MD, Fogel HM (1991). Maxillary molars with two palatal roots: a retrospective clinical study. J Endod.

[32] Crincoli V, Di Bisceglie MB, Scivetti M, Favia A, Di Comite M (2010). Dens invaginatus: a qualitative-quantitative analysis Case report of an upper second molar. Ultrastruct Pathol.

[33] Crosby KO, Barkhordar RA (1986). The multiple root canal system in a maxillary second molar A case report. Quintessence Int.

[34] de Almeida-Gomes F, Maniglia-Ferreira C, dos Santos RA (2007). Two palatal root canals in a maxillary second molar. Aust Endod J.

[35] Deveaux E (1999). Maxillary second molar with two palatal roots. J Endod.

[36] Fahid A, Taintor JF (1988). Maxillary second molar with three buccal roots. J Endod.

[37] Fava LR (1980). [Endodontic therapy in an abnormal case Maxillary second molar with four roots]. Rev Assoc Paul Cir Dent.

[38] Ghoddusi J, Mesgarani A, Gharagozloo S (2008). Endodontic re-treatment of maxillary second molar with two separate palatal roots: a case report. Iran Endod J.

[39] Grossman KE (1981). Endodontics involving an unusual case of fusion. J Endod.

[40] Hans MK, Chander S, Ahluwalia AS, Chinna H (2015). Non syndromic bilateral microdontia of maxillary second molars: a very rare finding. J Clin Diagn Res.

[41] Herrero Moraes S, Correa Costa ME, Carlos Ribeiro J (1988). [A maxillary second molar with five canals]. Dens (Curitiba).

[42] Holderrieth S, Gernhardt CR (2009). Maxillary molars with morphologic variations of the palatal root canals: a report of four cases. J Endod.

[43] Ioannidis K, Lambrianidis T, Beltes P, Besi E, Malliari M (2011). Endodontic management and cone-beam computed tomography evaluation of seven maxillary and mandibular molars with single roots and single canals in a patient. J Endod.

[44] Jafarzadeh H, Javidi M, Zarei M (2006). Endodontic retreatment of a maxillary second molar with three separate buccal roots. Aust Endod J.

[45] Jaikrishnan S, Kottoor J, Mathew J, Kumar SR, George S, Hari K (2015). Evaluation and endodontic management of a patient with 6 single-rooted molars: a case report. Gen Dent.

[46] Kaplowitz GJ (1983). Unusual canal anatomy in the distobuccal root of a maxillary second molar. Clin Prev Dent.

[47] Kim JR, Choi SB, Park SH (2008). A maxillary second molar with 6 canals: a case report. Quintessence Int.

[48] Kotoku K, Matsumoto Y, Aoki K (1978). [A case of the upper first molar with two roots and second molar with one root (author's transl)]. Shikwa Gakuho.

[49] Ma L, Yu J, Sun JJ (2011). [Maxillary first molar with three mesiobuccal root canals: a case report]. Hua Xi Kou Qiang Yi Xue Za Zhi.

[50] Malagnino V, Gallottini L, Passariello P (1997). Some unusual clinical cases on root anatomy of permanent maxillary molars. J Endod.

[51] Morinaga K, Aida N, Asai T, Tezen C, Ide Y, Nakagawa K (2010). Dens evaginatus on occlusal surface of maxillary second molar: a case report. Bull Tokyo Dent Coll.

[52] Ozcan E, Aktan AM, Ari H (2009). A case report: Unusual anatomy of maxillary second molar with 3 mesiobuccal canals. Oral Surg Oral Med Oral Pathol Oral Radiol Endod.

[53] Pasternak Junior B, Teixeira CS, Silva RG, Vansan LP, Sousa Neto MD (2007). Treatment of a second maxillary molar with six canals. Aust Endod J.

[54] Patel S, Patel P (2012). Endodontic management of maxillary second molar with two palatal roots: a report of two cases. Case Rep Dent.

[55] Paul B, Dube K (2013). Endodontic treatment of a maxillary second molar with two separate palatal roots: a case report. J Clin Diagn Res.

[56] Prashanth MB, Jain P, Patni P (2010). Maxillary right second molar with two palatal root canals. J Conserv Dent.

[57] Qun L, Longing N, Qing Y, Yuan L, Jun W, Qingyue D (2009). A case of asymmetric maxillary second molar with double palatal roots. Quintessence Int.

[58] Radwan A, Kim SG (2014). Treatment of a hypertaurodontic maxillary second molar in a patient with 10 taurodonts: a case report. J Endod.

[59] Rome WJ (1984). Endodontic therapy involving an unusual case of gemination. J Endod.

[60] Scarparo RK, Pereira L, Moro D, Grundling G, Gomes M, Grecca FS (2011). Morphologic variations of maxillary molars palatal root and the importance of its knowledge for endodontic practice: a case series. J Contemp Dent Pract.

[61] Sert S, Bayrl G (2004). Taurodontism in six molars: a case report. J Endod.

[62] Shin SJ, Park JW, Lee JK, Hwang SW (2007). Unusual root canal anatomy in maxillary second molars: two case reports. Oral Surg Oral Med Oral Pathol Oral Radiol Endod.

[63] Shojaeian S, Ghoddusi J, Hajian S (2013). A case report of maxillary second molar with two palatal root canals and a furcal enamel pearl. Iran Endod J.

[64] Simsek N, Keles A, Bulut ET (2013). Unusual root canal morphology of the maxillary second molar: a case report. Case Rep Dent.

[65] Thompson BH (1988). Endodontic therapy of an unusual maxillary second molar. J Endod.

[66] Wang Y, Hui X, Huang DM (2011). [Maxillary second molar with curved single root and single canal: a case report]. Hua Xi Kou Qiang Yi Xue Za Zhi.

[67] Weinstein T, Rosano G, Del Fabbro M, Taschieri S (2010). Endodontic treatment of a geminated maxillary second molar using an endoscope as magnification device. Int Endod J.

[68] Zhang QF, Liu GQ, Zhao HJ, Chen J, Zhang XH (2009). [Maxillary second molar with two buccal and two lingual root canals: a case report]. Hua Xi Kou Qiang Yi Xue Za Zhi.

[69] Zhang TT, Qiu W, Ming CX (2010). [Maxillary second molar with two palatal root canals: a case report]. Hua Xi Kou Qiang Yi Xue Za Zhi.

[70] Zhu Z, Zhao SL (2011). [Maxillary second molar with five root canals: a case report]. Shanghai Kou Qiang Yi Xue.

[71] Zmener O, Peirano A (1998). Endodontic therapy in a maxillary second molar with three buccal roots. J Endod.

[72] Ng YL, Aung TH, Alavi A, Gulabivala K (2001). Root and canal morphology of Burmese maxillary molars. Int Endod J.

[73] Rwenyonyi CM, Kutesa AM, Muwazi LM, Buwembo W (2007). Root and canal morphology of maxillary first and second permanent molar teeth in a Ugandan population. Int Endod J.

[74] Rouhani A, Bagherpour A, Akbari M, Azizi M, Nejat A, Naghavi N (2014). Cone-beam computed tomography evaluation of maxillary first and second molars in Iranian population: a morphological study. Iran Endod J.

[75] Silva EJ, Nejaim Y, Silva AI, Haiter-Neto F, Zaia AA, Cohenca N (2014). Evaluation of root canal configuration of maxillary molars in a Brazilian population using cone-beam computed tomographic imaging: an in vivo study. J Endod.

[76] Libfeld H, Rotstein I (1989). Incidence of four-rooted maxillary second molars: literature review and radiographic survey of 1,200 teeth. J Endod.

[77] Peikoff MD, Christie WH, Fogel HM (1996). The maxillary second molar: variations in the number of roots and canals. Int Endod J.

[78] Versiani MA, Pecora JD, de Sousa-Neto MD (2012). Root and root canal morphology of four-rooted maxillary second molars: a micro-computed tomography study. J Endod.

[79] al Shalabi RM, Omer OE, Glennon J, Jennings M, Claffey NM (2000). Root canal anatomy of maxillary first and second permanent molars. Int Endod J.

[80] Li L, Zhan FL, Jin YW (2014). [Preliminary study on root canal morphology of maxillary second molars]. Shanghai Kou Qiang Yi Xue.

[81] Singh S, Pawar M (2015). Root canal morphology of South Asian Indian maxillary molar teeth. Eur J Dent.

[82] Al-Fouzan KS, Ounis HF, Merdad K, Al-Hezaimi K (2013). Incidence of canal systems in the mesio-buccal roots of maxillary first and second molars in Saudi Arabian population. Aust Endod J.

[83] Domark JD, Hatton JF, Benison RP, Hildebolt CF (2013). An ex vivo comparison of digital radiography and cone-beam and micro computed tomography in the detection of the number of canals in the mesiobuccal roots of maxillary molars. J Endod.

[84] Reis AG, Grazziotin-Soares R, Barletta FB, Fontanella VR, Mahl CR (2013). Second canal in mesiobuccal root of maxillary molars is correlated with root third and patient age: a cone-beam computed tomographic study. J Endod.

[85] Silveira LF, Marques MM, da Costa RK, Martos J, Lorenzi A (2013). Location and negotiability of second mesiobuccal canal in upper molar by tomographic and anatomical macroscopic analysis. Surg Radiol Anat.

[86] Vizzotto MB, Silveira PF, Arus NA, Montagner F, Gomes BP, da Silveira HE (2013). CBCT for the assessment of second mesiobuccal (MB2) canals in maxillary molar teeth: effect of voxel size and presence of root filling. Int Endod J.

[87] Bauman R, Scarfe W, Clark S, Morelli J, Scheetz J, Farman A (2011). Ex vivo detection of mesiobuccal canals in maxillary molars using CBCT at four different isotropic voxel dimensions. Int Endod J.

[88] Lee JH, Kim KD, Lee JK, Park W, Jeong JS, Lee Y, Gu Y, Chang SW, Son WJ, Lee WC, Baek SH, Bae KS, Kum KY (2011). Mesiobuccal root canal anatomy of Korean maxillary first and second molars by cone-beam computed tomography. Oral Surg Oral Med Oral Pathol Oral Radiol Endod.

[89] Degerness RA, Bowles WR (2010). Dimension, anatomy and morphology of the mesiobuccal root canal system in maxillary molars. J Endod.

[90] Gao Y, An SF, Ling JQ (2006). [An in vitro study on the incidence of the second mesiobuccal canal in the mesiobuccal root of the first and second maxillary molars]. Zhonghua Kou Qiang Yi Xue Za Zhi.

[91] Shahravan A, Rekabi A, Shahabi H, Ashuri R, Mirzazadeh A, Rad M, Haghani J (2010). A digital stereomicroscopic study of the furcation wall thickness of mesiobuccal roots of maxillary first and second molars. Iran Endod J.

[92] Zhao YM, Xu X, Sun J, Qiang YL, Qi QG (2009). [In vitro study of the secondary mesiobuccal canal of the maxillary second molar]. Hua Xi Kou Qiang Yi Xue Za Zhi.

[93] Wang WZ (2004). [Location and dilation of the second mesiobuccal canals in maxillary molars]. Shanghai Kou Qiang Yi Xue.

[94] Wolcott J, Ishley D, Kennedy W, Johnson S, Minnich S (2002). Clinical investigation of second mesiobuccal canals in endodontically treated and retreated maxillary molars. J Endod.

[95] Wolcott J, Ishley D, Kennedy W, Johnson S, Minnich S, Meyers J (2005). A 5 yr clinical investigation of second mesiobuccal canals in endodontically treated and retreated maxillary molars. J Endod.

[96] Wolcott J, Minnich S, Ishley D, Kennedy W, Johnson S (2002). Second mesiobuccal canals in maxillary molars: their incidence and importance. Compend Contin Educ Dent.

[97] Zhang CF, Ding RY, Yin XZ, Zhao BH, Lin QG (2003). [Location and negotiation of second mesiobuccal canals in maxillary molars]. Zhonghua Kou Qiang Yi Xue Za Zhi.

[98] Buhrley LJ, Barrows MJ, BeGole EA, Wenckus CS (2002). Effect of magnification on locating the MB2 canal in maxillary molars. J Endod.

[99] Schwarze T, Baethge C, Stecher T, Geurtsen W (2002). Identification of second canals in the mesiobuccal root of maxillary first and second molars using magnifying loupes or an operating microscope. Aust Endod J.

[100] Sempira HN, Hartwell GR (2000). Frequency of second mesiobuccal canals in maxillary molars as determined by use of an operating microscope: a clinical study. J Endod.

[101] Stropko JJ (1999). Canal morphology of maxillary molars: clinical observations of canal configurations. J Endod.

[102] Eskoz N, Weine FS (1995). Canal configuration of the mesiobuccal root of the maxillary second molar. J Endod.

[103] Gilles J, Reader A (1990). An SEM investigation of the mesiolingual canal in human maxillary first and second molars. Oral Surg Oral Med Oral Pathol.

[104] Singh C, Sikri VK, Arora R (1994). Study of root canals and their configuration in maxillary second permanent molar. Indian J Dent Res.

[105] Weng XL, Yu SB, Zhao SL, Wang HG, Mu T, Tang RY, Zhou XD (2009). Root canal morphology of permanent maxillary teeth in the Han nationality in Chinese Guanzhong area: a new modified root canal staining technique. J Endod.

[106] Karaman GT, Onay EO, Ungor M, Colak M (2011). Evaluating the potential key factors in assessing the morphology of mesiobuccal canal in maxillary first and second molars. Aust Endod J.

[107] Yang B, Lu Q, Bai QX, Zhang Y, Liu XJ, Liu ZJ (2013). [Evaluation of the prevalence of the maxillary molars with two palatal roots by cone-beam CT]. Zhonghua Kou Qiang Yi Xue Za Zhi.

[108] Plotino G, Tocci L, Grande NM, Testarelli L, Messineo D, Ciotti M, Glassman G, D'Ambrosio F, Gambarini G (2013). Symmetry of root and root canal morphology of maxillary and mandibular molars in a white population: a cone-beam computed tomography study in vivo. J Endod.

[109] Kim SY, Yang SE (2012). Cone-beam computed tomography study of incidence of distolingual root and distance from distolingual canal to buccal cortical bone of mandibular first molars in a Korean population. J Endod.

[110] Marroquin BB, El-Sayed MA, Willershausen-Zonnchen B (2004). Morphology of the physiological foramen: I Maxillary and mandibular molars. J Endod.

[111] Degerness R, Bowles W (2008). Anatomic determination of the mesiobuccal root resection level in maxillary molars. J Endod.

[112] Deutsch AS, Musikant BL (2004). Morphological measurements of anatomic landmarks in human maxillary and mandibular molar pulp chambers. J Endod.

[113] Jafarzadeh H, Wu YN (2007). The C-shaped root canal configuration: a review. J Endod.

[114] Yang ZP, Yang SF, Lin YC, Shay JC, Chi CY (1988). C-shaped root canals in mandibular second molars in a Chinese population. Endod Dent Traumatol.

[115] Burklein S, Breuer D, Schafer E (2011). Prevalence of taurodont and pyramidal molars in a German population. J Endod.

[116] Imura N, Hata GI, Toda T, Otani SM, Fagundes MI (1998). Two canals in mesiobuccal roots of maxillary molars. Int Endod J.

[117] Abarca J, Zaror C, Monardes H, Hermosilla V, Munoz C, Cantin M (2014). Morphology of the Physiological Apical Foramen in Maxillary and Mandibular. First Molars.

[118] Prakash R, Bhargavi N, Rajan J, Joseph R, Velmurugan N, Kandaswamy D (2007). MB2 in maxillary second molar. Indian J Dent Res.

[119] Corcoran J, Apicella MJ, Mines P (2007). The effect of operator experience in locating additional canals in maxillary molars. J Endod.

[120] Mines P, Loushine RJ, West LA, Liewehr FR, Zadinsky JR (1999). Use of the microscope in endodontics: a report based on a questionnaire. J Endod.

[121] Gorduysus MO, Gorduysus M, Friedman S (2001). Operating microscope improves negotiation of second mesiobuccal canals in maxillary molars. J Endod.

[122] Sert S, Bayirli GS (2004). Evaluation of the root canal configurations of the mandibular and maxillary permanent teeth by gender in the Turkish population. J Endod.

[123] Omer OE, Al Shalabi RM, Jennings M, Glennon J, Claffey NM (2004). A comparison between clearing and radiographic techniques in the study of the root-canal anatomy of maxillary first and second molars. Int Endod J.

[124] Mirmohammadi H, Mahdi L, Partovi P, Khademi A, Shemesh H, Hassan B (2015). Accuracy of Cone-beam Computed Tomography in the Detection of a Second Mesiobuccal Root Canal in Endodontically Treated Teeth: An Ex Vivo Study. J Endod.

[125] Ramamurthy R, Scheetz JP, Clark SJ, Farman AG (2006). Effects of imaging system and exposure on accurate detection of the second mesio-buccal canal in maxillary molar teeth. Oral Surg Oral Med Oral Pathol Oral Radiol Endod.

[126] Ibarrola JL, Knowles KI, Ludlow MO, McKinley IB Jr (1997). Factors affecting the negotiability of second mesiobuccal canals in maxillary molars. J Endod.

[127] Martos J, Tatsch GH, Tatsch AC, Silveira LF, Ferrer-Luque CM (2011). Anatomical evaluation of the root canal diameter and root thickness on the apical third of mesial roots of molars. Anat Sci Int.

